# Mental disorders and suicidal behavior in refugees and Swedish-born individuals: is the association affected by work disability?

**DOI:** 10.1007/s00127-019-01824-5

**Published:** 2020-01-02

**Authors:** Emma Björkenstam, Magnus Helgesson, Ridwanul Amin, Theis Lange, Ellenor Mittendorfer-Rutz

**Affiliations:** 1 Division of Insurance Medicine, Department of Clinical Neuroscience, Karolinska Institutet, 171 77 Stockholm, Sweden; 2 Department of Community Health Sciences, Fielding School of Public Health and California Center for Population Research, University of California Los Angeles, Los Angeles, CA USA; 3 Department of Neuroscience, Psychiatry, Uppsala University, Uppsala, Sweden; 4 Section of Biostatistics, University of Copenhagen, Copenhagen, Denmark; 5 Center for Statistical Science, Peking University, Beijing, China

**Keywords:** Suicide, Self-harm, Migration, Refugees, Cohort, Sweden, Epidemiology, Sick leave, Disability pension, Work disability, Mental disorders

## Abstract

**Background:**

Among potential pathways to suicidal behavior in individuals with mental disorders (MD), work disability (WD) may play an important role. We examined the role of WD in the relationship between MD and suicidal behavior in Swedish-born individuals and refugees.

**Methods:**

The study cohort consisted of 4,195,058 individuals aged 16–64, residing in Sweden in 2004–2005, whereof 163,160 refugees were followed during 2006–2013 with respect to suicidal behavior. Risk estimates were calculated as hazard ratios (HR) with 95% confidence intervals (CI). The reference groups comprised individuals with neither MD nor WD. WD factors (sickness absence (SA) and disability pension (DP)) were explored as potential modifiers and mediators.

**Results:**

In both Swedish-born and refugees, SA and DP were associated with an elevated risk of suicide attempt regardless of MD. In refugees, HRs for suicide attempt in long-term SA ranged from 2.96 (95% CI: 2.14–4.09) (no MD) to 6.23 (95% CI: 3.21–12.08) (MD). Similar associations were observed in Swedish-born. Elevated suicide attempt risks were also observed in DP. In Swedish-born individuals, there was a synergy effect between MD, and SA and DP regarding suicidal behavior. Both SA and DP were found to mediate the studied associations in Swedish-born, but not in refugees.

**Conclusion:**

There is an effect modification and a mediating effect between mental disorders and WD for subsequent suicidal behavior in Swedish-born individuals. Also for refugees without MD, WD is a risk factor for subsequent suicidal behavior. Particularly for Swedish-born individuals with MD, information on WD is vital in a clinical suicide risk assessment.

**Electronic supplementary material:**

The online version of this article (10.1007/s00127-019-01824-5) contains supplementary material, which is available to authorized users.

## Introduction

There has been a dramatic increase in migration globally over the last decade [1]. In 2017, there were 258 million migrants worldwide, including 25.9 million refugees and asylum seekers [1]. Sweden is one of the largest European recipients of refugees, with more than 160,000 individuals seeking asylum in 2015 alone [2].

Studies have shown that refugees have an increased risk of mental disorders in terms of post-traumatic stress disorder (PTSD), anxiety and depressive disorders [3–6]. Given these higher levels of psychiatric morbidity, it has further been hypothesized that refugees have an elevated risk of suicidal behavior, in terms of suicide attempt and suicide. However, there are hardly any studies examining this association [7–9]. One Danish study that investigated differences in injury mortality in refugees and immigrants reported lower rates of suicide [7] in refugees in comparison with the host Danish population. Studies on migrants not discriminating between refugees and non-refugee populations have shown higher rates of suicide attempt and lower rates of suicide compared with the respective host populations [10, 11].

Mental disorders have been pointed out as important risk factors for both suicide attempt and suicide [12–16]. In fact, it has been shown that as many as 90% of individuals who die by suicide have been diagnosed with a mental disorder prior to their death [13]. In general, depressive disorders are the mental disorders most strongly associated with suicide attempt and suicide, followed by substance abuse and schizophrenia [12–14]. Studies have reported geographical and cultural differences with respect to prevalence of mental disorders in suicide victims [17, 18]. Still, to which extent mental disorders affect suicide attempt and suicide in refugees remains unknown to date.

When studying mental disorders and suicidal behavior in refugees, another aspect to consider is the economic welfare of the country studied, including opportunities for immigrants to participate in the labor market. Studies have shown that mental disorders have a strong effect on occupational functioning and work capacity [19], and people with mental disorders face a higher risk of work disability (WD), often conceptualized as sickness absence (SA) and disability pension (DP) [20–22]. As opportunities for refugees to participate in the labor market might be different than opportunities for those in the host population, the effects of mental disorders on WD might affect these groups differently. A recent Swedish study examining the risk of labor market marginalization (LMM), i.e. severe problems in finding and keeping a job, in refugees, showed that refugees in general had an elevated risk of LMM when compared to the Swedish-born population [23]. According to another Swedish study examining the associations between mental disorders and LMM, among young adults aged 20–35 years with mental disorders, all immigrants had a lower risk of SA and DP, compared with the Swedish-born population [20]. Whether similar associations hold for refugees is unclear to date.

WD may play an important role in the relationship between mental disorder and suicide attempt and suicide. Several studies have shown associations between mental disorders and SA [21, 22, 24], that in turn is a risk factor for suicidal behavior [25–27]. A number of studies have shown that DP, due to mental disorders, is associated with both suicide attempt and suicide [28–30]. Moreover, DP has been demonstrated as a strong risk indicator for suicidal behavior among certain groups, including multiple sclerosis (MS) [31]. Whether this also holds in patients with mental disorders remains unknown.

On the other hand, WD may also act as a mediating link between mental disorders and suicidal behavior, as exclusion from the labor market might lead to marginalization, social isolation and loss of purpose, conditions that are detrimental to suicidal behavior [32, 33]. Still to date, research on the potential mediating role of SA and DP in the association between mental disorder and subsequent suicide attempt and suicide is missing. Moreover, if these associations differ between refugees and Swedish-born individuals is yet to be explored. To prevent suicidal behavior in patients with mental disorders, studies investigating potential mechanisms, such as WD, linking mental disorders to suicidal behavior are essential, yet lacking.

The current register-based study used a large cohort of nearly 4.2 million individuals, whereof over 163,000 refugees, to examine the association between mental disorders, WD, and subsequent suicide attempt and suicide in refugees and Swedish-born individuals. In addition, we investigated if the effect of mental disorders on suicide attempt and suicide was modified or mediated by WD.

## Methods

### Study population

The study population was defined as all individuals, aged 16–64 years, residing in Sweden on December 31st, 2004 (*n* = 5,750,669), who were also residing in Sweden on December 31st, 2005 (*n* = 5,709,767). Only those with complete information on their reason for settlement in Sweden were included (*n* = 5,488,075). Further, as we compared refugees with Swedish-born individuals, non-refugee immigrants (*n* = 429,666) were excluded.

Lastly, in an attempt to avoid reverse causation, individuals who were either on SA for more than 90 days, or on DP during the period 2000 through 2004 were excluded (*n* = 863,351). Thus, the final study population included 4,195,058 individuals, whereof 163,160 refugees.

The unique (de-identified) Swedish personal identity number was used to link the cohort to multiple health care and administrative registers [34]. The Longitudinal Integration Database for Health Insurance and Labor Market Studies (LISA) includes existing data from the labor market, educational and social sectors. The STATIV register holds migration-related information, including reasons for settlement, e.g. refugee status. The National Patient Register (NPR) includes all individuals admitted to psychiatric and general hospitals, with complete coverage for inpatient care since 1987 and for specialized outpatient care since 2001. The Causes of Death Register (CDR) comprises information on all deaths of Swedish residents since 1952. Diagnoses in NPR and CDR are coded according to the International Classification of Diseases and Related Health Problems version 10 (ICD-10).

### Measures

#### Mental disorders

Mental disorders were defined as having a psychiatric diagnosis during psychiatric in- or specialized outpatient care, as recorded in the NPR. All hospitalizations and specialized outpatient visits during the period 2000–2004, i.e. 5 years prior to baseline, were collected.

#### Suicide attempt and suicide

The individuals were prospectively followed up from January 1st, 2006 until December 31st, 2013, with respect to suicide attempt and suicide. Suicide attempt was defined as having received at least one diagnosis (ICD-10: X60-84 or Y10-34) in inpatient care, obtained from the NPR, during follow-up. Suicide was defined as having an underlying cause of death in the Causes of Death Register, coded according to the ICD-10 (X60-84 or Y10-34). With our definition of suicide attempt and suicide including events with undetermined intent, our aim was to reduce spatial and secular trends in detecting and classifying cases of suicidal behavior [35].

#### Refugee status

According to the Geneva Convention on Refugees, a refugee is a person who is outside her/his country of citizenship because of well-founded grounds for fear of persecution, and is unable to obtain sanctuary from the home country or, owing to such fear, is unwilling to avail himself for the protection of that country [36]. In the current study, a refugee was defined as an individual receiving a residence permit in Sweden as a refugee, or an individual who is being granted residence permit due to “in need of protection” or on “humanitarian grounds”.

#### Sickness absence and disability pension

Captured in 2005, the WD measures, based on medical assessment, included SA and DP. In Sweden, all residents aged 16–65 years, with income from work, unemployment benefits, or student benefits covered by the national sickness absence insurance regime and can be sickness absent with benefits if unable to work due to disease or injury. For those employed, the employer usually pays for the first 14 days of an SA spell. Thus, data on most of the short SA spells are not available. For individuals aged 30–64 years, DP can be granted to those who due to disease or injury have a permanently impaired work capacity. Individuals between 19 and 29 years can be granted time-restricted DP if work capacity is reduced, or if compulsory education is not completed before 19 years of age. We used the number of net SA days and created the following categories: no SA days, short-term SA (1–90 days) and long-term SA (> 90 days). For DP, a dichotomous variable was created for “DP in 2005”.

#### Potential confounders

Several confounding factors, measured in 2004, were considered. Age and sex were included in the analyses. We controlled for highest attained educational level, family situation and type of residential area (please see Table 1 for information on categorization of the confounders). Missing values for a covariate were categorized in separate categories. History of suicide attempt, a known risk factor for subsequent attempt and suicide, was defined as at least one hospitalization for suicide attempt in 2000–2004. Last, adjustments were made for somatic comorbidity, defined as inpatient or specialized outpatient care in 2000–2004 with a main diagnosis for somatic disease. Four dichotomized groups were created: diseases of the musculoskeletal system and connective tissue (ICD-10: M00–M99), neoplasms (ICD-10: C00–C97, D10–D48), diseases of the circulatory system (ICD-10: I00–I99), and other somatic disorders (the rest of ICD-10 codes except for F-codes).

**Table 1:** Cohort characteristics stratified by work disability, in Swedish-born individuals and refugees, aged 16–64 years residing in Sweden in 2004 and 2005

Cohort characteristics	Swedish-born individuals			Refugees		
	All	No work disability^c^	Work disability^c^	All	No work disability^c^	Work disability^c^
All, (*n*, row percent)	4,031,898 (100)	3,680,255 (91)	351,643 (9)	163,160 (100)	147,688 (91)	15,472 (10)
Sociodemographic factors^a^						
Sex						
Women	1,873,273 (46)	1,664,598 (45)	208,675 (59)	67,791 (42)	60,463 (41)	7328 (47)
Men	2,158,625 (54)	2,015,657 (55)	142,968 (41)	95,369 (58)	87,225 (59)	8144 (53)
Age (years)						
16–24	821,002 (20)	791,813 (22)	29,189 (8)	40,683 (25)	39,289 (27)	1394 (9)
25–34	861,608 (21)	782,303 (21)	79,305 (23)	35,482 (22)	32,160 (22)	3322 (21)
35–44	872,702 (22)	789,222 (21)	83,480 (24)	50,041 (31)	44,271 (30)	5770 (37)
45–54	771,178 (19)	690,677 (19)	80,501 (23)	28,794 (18)	24,741 (17)	4053 (26)
55–64	705,408 (17)	626,240 (17)	79,168 (23)	8160 (5)	7227 (5)	933 (6)
Educational level (years)						
Compulsory school (< 9)	807,608 (20)	746,850 (20)	60,758 (17)	47,063 (29)	43,537 (29)	3526 (23)
High school (10–12)	1,887,346 (47)	1,694,895 (46)	192,451 (55)	63,601 (39)	56,041 (38)	7560 (49)
College or university (> 12)	1,319,631 (33)	1,222,063 (33)	97,568 (28)	42,225 (26)	38,260 (26)	3965 (26)
Missing	17,313 (0)	16,447 (0)	866 (0)	10,271 (6)	9850 (7)	421 (3)
Family situation						
Married/cohabiting without children^d^	551,439 (14)	490,140 (13)	61,299 (17)	11,285 (7)	9850 (7)	1435 (9)
Married/living with partner with children^d^	1,374,023 (34)	1,238,870 (34)	135,153 (38)	68,865 (42)	60,407 (41)	8458 (55)
Single/divorced/separated/widowed without children^d^	1,462,003 (36)	1,343,300 (37)	118,703 (34)	50,813 (31)	46,919 (32)	3894 (25)
Single/divorced/separated/widowed with children^d^	213,157 (5)	183,024 (5)	30,133 (9)	12,004 (7)	10,532 (7)	1472 (10)
Children (≤ 20 years old) living at home	431,272 (11)	424,917 (12)	6355 (2)	20,188 (12)	19,975 (14)	213 (1)
Missing	< 10^e^ (0)	< 10^e^ (0)	< 10^e^ (0)	< 10^e^ (0)	< 10^e^ (0)	< 10^e^ (0)
Place of residence						
Big city area	1,418,613 (35)	1,303,802 (35)	114,811 (33)	77,859 (48)	70,194 (48)	7665 (50)
Intermediate (> 90,000 inhabitants)	1,458,708 (36)	1,331,980 (36)	126,728 (36)	59,380 (36)	54,026 (37)	5354 (35)
Small (rural municipalities)	1,154,577 (29)	1,044,473 (28)	110,104 (31)	25,921 (16)	23,468 (16)	2453 (16)
Mental disorders and health-related factors^b^						
No mental disorder	3,942,858 (98)	3,605,515 (98)	337,343 (96)	156,123 (96)	141,538 (96)	14,585 (94)
Mental disorder	89,040 (2)	74,740 (2)	14,300 (4)	7037 (4)	6150 (4)	887 (6)
History of suicide attempt	9831 (0)	6460 (0)	3371 (1)	804 (0)	528 (0)	276 (2)
Somatic comorbidity						
Neoplasms	184,711 (5)	161,140 (4)	24,571 (7)	5285 (3)	4420 (3)	865 (6)
Cardiovascular disease	129,664 (3)	109,774 (3)	19,890 (6)	5294 (3)	4415 (3)	879 (6)
Musculoskeletal disorders	297,058 (7)	251,825 (7)	45,233 (13)	12,989 (8)	10,970 (7)	2019 (13)
Other somatic disorders	1,845,811 (46)	1,632,754 (44)	213,057 (61)	83,583 (51)	73,653 (50)	9930 (64)
Outcome						
Suicide attempt (rate per 100,000 person-years)	19,940 (62.9)	16,507 (57.0)	3433 (125.1)	1056 (83.7)	925 (81.1)	131 (108.2)
Suicide (rate per 100,000 person-years)	4576 (14.4)	3841 (13.2)	735 (26.6)	146 (11.5)	120 (10.5)	26 (21.4)

^a^In 2004

^b^In 2000–2004

^c^Either long-term sickness absence or disability pension in 2005

^d^Living at home

^e^For ethical reasons, i.e. the risk of identification of individuals, if the number of suicides is < 10, it is not reported

### Statistical analyses

We used Cox regression to statistically evaluate the associations between mental disorders and each outcome, respectively, presented as hazard ratios (HR) with 95% confidence intervals (CI). We assessed person-years at risk by totaling the years that the individuals were living in Sweden during the follow-up period. The entry date was defined as January 1st, 2006, and the exit date as the date of inpatient care for suicide attempt (outcome suicide attempt), date of suicide (outcome suicide), date of death any cause (in the analyses with suicide attempt as outcome measure), date of death from other causes (outcome suicide), date of emigration, or the end of follow-up (December 31st, 2013). In the regression models, mental disorders and WD were treated as non-time-varying binary data.

We examined the associations between mental disorders and each outcome in one crude and two multi-adjusted regression models. Model 2 was adjusted for age, sex, education, family situation and place of residence. Model 3 was further adjusted for prior suicide attempt and somatic comorbidity. The reference groups comprised individuals with no mental disorder.

For evaluating biological interaction [37] between mental disorders and SA and DP, respectively, synergy indices (SI) were calculated. SI measures the interaction, expressed as the ratio of the relative excess risk for the combined effect of the risk factors and the sum of the relative excess risks for each separate effect of the two risk factors. An SI greater than 1 indicates that the absolute excess risk for those exposed to both risk factors is greater than the sum of the absolute excess risks for those exposed to each separate risk factor [38]. In the SI analysis, we merged individuals with ≥ 1 SA day to one group.

To test the potential mediation of the effect of mental disorder on suicide attempt and suicide by SA and DP, a mediation analysis was conducted using a SAS macro for causal mediation analysis with survival data, provided by Valeri et al. [39]. This is an extension of the Baron and Kenny method [40], in which a regression model examining the association between the proposed mediator (M) and the independent variable (IV) is compared with a regression model examining the association between the dependent variable (DV) and the IV together with the proposed M. A counterfactual framework, which allows for interactions between the IV and M, is then used to compare the two models to estimate the direct effect of the IV on DV and the indirect effect of the IV on DV via the proposed M [41]. Comparison of the magnitude of the direct and indirect effect allows for the estimation of the proportion of total effect that is mediated. The results are presented as the direct effect of mental disorder on the studied outcomes, the indirect effect of mental disorder on outcomes mediated by SA and DP, respectively, and the estimated total effect representing the combined natural direct and indirect effect.

#### Sensitivity analyses

We conducted a sensitivity analysis, in which individuals who were granted residence permits due to ‘in need of protection’ and on ‘humanitarian grounds’ were excluded from the refugee category. Additionally, for both suicide attempt and suicide, we conducted separate analyses for events coded as undetermined intent.

## Results

Table 1 shows that 9% of Swedish-born individuals and 10% of refugees were either on long-term SA or DP in 2005. Compared to Swedish-born individuals, refugees were slightly younger, had lower levels of education, and lived in larger cities to a greater extent (Table 1). Two percent of Swedish-born and 4% of refugees were treated at least once in 2000–2004 with mental disorder as the main diagnosis. Compared to those without WD, it was more common among those with WD (both Swedish-born and refugees) to be slightly older, have a history of suicide attempt, and have a somatic comorbidity. In both Swedish-born and refugees, mental disorders were associated with both long-term SA and DP (data not shown).

During the follow-up, 19,940 Swedish-born individuals (62.9 per 100,000 person-years) and 1056 refugees (83.7 per 100,000 person-years) were treated for suicide attempt (Table 1). For both Swedish-born and refugees, the rates of suicide attempt were higher in those on WD in comparison with those without WD. In total, 4576 Swedish-born (14.4 per 100,000 person-years) and 146 refugees (11.5 per 100,000 person-years) died by suicide (Table 1). Swedish-born and refugees on WD had higher suicide rates during the follow-up period compared to those with no WD.

Table 2 presents crude and multi-adjusted hazard ratios (HR) with 95% confidence intervals (CI) for suicide attempt by mental disorders and WD factors in Swedish-born individuals and refugees, respectively. In both Swedish-born and refugees, long-term SA and DP were associated with elevated risk of suicide attempt in individuals with and without mental disorders. In refugees, compared to those with no mental disorder, the multi-adjusted HR for suicide attempt in long-term SA without mental disorder was 2.96 (95% CI: 2.14–4.09), and in long-term SA with mental disorder 6.23 (95% CI: 3.21–12.08). In Swedish-born individuals, a dose–response relationship was observed for SA length and risk of suicide attempt, i.e. the risk increased as the number of SA days increased, in individuals with and without mental disorders. Elevated suicide attempt risks were also observed in DP, where both refugees and Swedish-born individuals on DP, with and without mental disorders, had a markedly elevated risk of suicide attempt, compared with individuals with no mental disorder and no DP. Refugees with a mental disorder, on DP, had a fivefold risk of suicide attempt (multi-adjusted HR: 5.46 (95% CI 2.99–9.99)) and Swedish-born a tenfold risk (multi-adjusted HR: 10.27 (95% CI 8.91–11.83)). The HRs decreased markedly but remained significant in the fully adjusted model (Table 2, Model 3), where sociodemographics and comorbid factors were included.

**Table 2:** Hazard ratios (HR) with 95% confidence intervals (CIs) for risk of suicide attempt by diagnosis of mental disorder and work disability factors in Swedish-born individuals and refugees

	Swedish-born individuals				Refugees			
	Suicide attempt *N* (rate per 100,000 person-years)	Model 1^a^	Model 2^b^	Model 3^c^	Suicide attempt *N* (rate per 100,000 person-years)	Model 1^a^	Model 2^b^	Model 3^c^
Sickness absence (SA) in 2005								
No mental disorder, no SA	12,804 (45.1)	1 (REF)	1 (REF)	1 (REF)	723 (65.8)	1 (REF)	1 (REF)	1 (REF)
No mental disorder, SA 1–90 days	1667 (78.1)	1.73 (1.65–1.82)	2.09 (1.99–2.21)	1.94 (1.84–2.04)	57 (68.6)	1.05 (0.80–1.37)	1.31 (1.00–1.72)	1.22 (0.93–1.60)
No mental disorder, SA > 90 days	736 (153.1)	3.39 (3.15–3.65)	4.27 (3.96–4.61)	3.88 (3.59–4.18)	40 (154.9)	2.36 (1.72–3.24)	3.21 (2.32–4.43)	2.96 (2.14–4.09)
Any mental disorder, no SA	3978 (690.2)	15.25 (14.72–15.81)	8.93 (8.60–9.27)	6.67 (6.41–6.94)	219 (450.4)	6.82 (5.87–7.94)	5.86 (5.03–6.83)	4.20 (3.55–4.96)
Any mental disorder, SA 1–90 days	458 (708.4)	15.65 (14.26–17.18)	13.74 (12.51–15.09)	9.94 (9.04–10.93)	< 10^d^ (254.4)	3.87 (1.93–7.76)	4.08 (2.03–8.20)	2.77 (1.38–5.60)
Any mental disorder, SA > 90 days	297 (1007.4)	22.25 (19.84–24.96)	19.63 (17.49–22.04)	14.43 (12.84–16.21)	< 10^d^ (510.1)	7.75 (4.02–14.96)	8.87 (4.58–17.14)	6.23 (3.21–12.08)
Disability pension (DP) in 2005								
No mental disorder, no DP	15,112 (48.8)	1 (REF)	1 (REF)	1 (REF)	811 (67.5)	1 (REF)	1 (REF)	1 (REF)
No mental disorder, DP	95 (242.4)	4.95 (4.05–6.06)	3.45 (2.82–4.23)	3.15 (2.57–3.86)	< 10^d^ (152.4)	2.26 (1.17–4.35)	2.67 (1.38–5.18)	2.32 (1.20–4.51)
Any mental disorder, no DP	4533 (689.4)	14.08 (13.62–14.55)	8.73 (8.43–9.03)	6.50 (6.25–6.75)	225 (436.4)	6.45 (5.56–7.47)	5.52 (4.75–6.41)	3.98 (3.38–4.68)
Any mental disorder, DP	200 (1541.9)	31.35 (27.27–36.04)	14.74 (12.81–16.96)	10.27 (8.91–11.83)	11 (557.8)	8.23 (4.54–14.92)	8.33 (4.58–15.13)	5.46 (2.99–9.99)

^a^Model 1: crude

^b^Model 2: adjusted for age, sex, educational level, family situation and place of residence

^c^Model 3: Model 2 with additional adjustments for history of suicide attempt and somatic comorbidity

^d^For ethical reasons i.e. the risk of identification of individuals, if the number of events is < 10, it is not reported

In the analyses where we examined associations between mental disorders, WD and suicide (Table 3), among refugees, the number of suicides in each sub-group was small and hence the CIs overlapped to a greater extent. Still, the pattern was similar to the results from the analyses of suicide attempt as outcome. In Swedish-born individuals, both SA and DP modified the association between mental disorder and suicide. Highest suicide risk was observed in those with mental disorders on long-term SA (multi-adjusted HR: 11.95 (95% CI 9.11–15.68)) or DP (multi-adjusted HR: 10.29 (95% CI 7.19–14.72)).

**Table 3:** Hazard ratios (HR) with 95% confidence intervals (CIs) for risk of suicide by diagnosis of mental disorder and work disability factors in Swedish-born individuals and refugees

	Swedish-born individuals				Refugees			
	Suicide *N* (rate per 100,000 person-years)	Model 1^a^	Model 2^b^	Model 3^c^	Suicide *N* (rate per 100,000 person-years)	Model 1^a^	Model 2^b^	Model 3^c^
Sickness absence (SA) in 2005								
No mental disorder, no SA	3259 (11.5)	1 (REF)	1 (REF)	1 (REF)	90 (8.2)	1 (REF)	1 (REF)	1 (REF)
No mental disorder, SA 1–90 days	395 (18.4)	1.61 (1.45–1.78)	1.85 (1.67–2.06)	1.81 (1.63–2.02)	15 (18.0)	2.20 (1.28–3.81)	2.48 (1.42–4.31)	2.32 (1.33–4.05)
No mental disorder, SA > 90 days	157 (32.4)	2.83 (2.42–3.33)	3.09 (2.63–3.63)	3.00 (2.55–3.52)	< 10^d^ (19.2)	2.35 (0.96–5.79)	2.58 (1.04–6.40)	2.34 (0.94–5.82)
Any mental disorder, no SA	626 (105.4)	9.21 (8.45–10.03)	7.85 (7.18–8.57)	6.72 (6.12–7.38)	32 (64.5)	7.90 (5.28–11.83)	8.02 (5.33–12.08)	6.03 (3.86–9.44)
Any mental disorder, SA 1–90 days	85 (127.5)	11.01 (8.45–13.67)	10.43 (8.39–12.97)	8.78 (7.05–10.94)	< 10^d^ (94.4)	11.55 (3.66–36.50)	13.01 (4.10–41.23)	10.22 (3.19–32.72)
Any mental disorder, SA > 90 days	54 (174.3)	15.23 (11.64–19.93)	14.07 (10.75–18.42)	11.95 (9.11–15.68)	< 10^d^ (55.5)	6.79 (0.95–48.72)	6.87 (0.95–49.45)	5.77 (0.80–41.65)
Disability pension (DP) in 2005								
No mental disorder, no DP	3,790 (12.2)	1 (REF)	1 (REF)	1 (REF)	110 (9.1)	1 (REF)	1 (REF)	1 (REF)
No mental disorder, DP	21 (53.1)	4.35 (2.84–6.69)	3.48 (2.27–5.36)	3.32 (2.16–5.11)	< 10^d^ (0.0)	N/A	N/A	N/A
Any mental disorder, no DP	734 (108.3)	8.87 (8.19–9.60)	7.68 (7.08–8.33)	6.54 (6.00–7.13)	34 (64.7)	7.09 (4.83–10.41)	7.20 (4.87–10.63)	5.49 (3.59–8.39)
Any mental disorder, DP	31 (221.9)	18.21 (12.79–25.92)	12.96 (9.09–18.49)	10.29 (7.19–14.72)	< 10^d^ (99.2)	10.87 (2.68–44.0)	10.72 (2.63–43.65)	6.78 (1.62–28.42)

^a^ Model 1: crude

^b^ Model 2: adjusted for age, sex, educational level, family situation and place of residence

^c^ Model 3: Model 2 with additional adjustments for history of suicide attempt and somatic comorbidity

^d^ For ethical reasons, i.e. the risk of identification of individuals, if the number of events is < 10, it is not reported

Evaluation of synergy effects between mental disorders and SA and DP (supplementary Tables 1, 2) revealed that, for Swedish-born, the risk of suicide attempt among those with a mental disorder who were on SA or on DP was higher than would be expected from the additive effect of the two exposures (multi-adjusted SI for analyses of SA: 1.55 (95% CI 1.42–1.68); and for DP (SI: 1.35 (95% CI 1.14–1.59)). Similar effects were observed for suicide, although the synergy effects between mental disorders and DP were not statistically significant. For refugees on the other hand, no statistically significant synergy effects were observed.

Mediation analyses were conducted for SA and DP, and for refugees and the Swedish-born population separately. The total effect of a mental disorder on both suicide attempt and suicide was partially mediated by SA in Swedish-born individuals but not in refugees (Table 4). This was indicated by a small but significant indirect effect of the mediated pathway. Moreover, in Swedish-born individuals, the proportion of the total effect mediated by SA was 7.0% for suicide attempt and 5.8% for suicide.

**Table 4:** Mediation analysis investigating the association of mental disorder with subsequent suicide attempt and suicide, mediated by sickness absence (SA) and disability pension (DP), respectively, Swedish-born individuals and refugees aged 16–64 years residing in Sweden in 2004 and 2005

Mediator and outcome	Direct effect (HR^a^, (95% CI)	Indirect effect (HR^a^, 95% CI)	Total effect (HR^a^, 95% CI)	Percentage of total effect mediated by SA and DP, respectively (%)
SA				
Suicide attempt				
Swedish-born individuals	9.57 (9.25–9.89)	1.07 (1.06–1.07)	10.21 (9.88–10.56)	7.0
Refugees	6.18 (5.34–7.14)	1.01 (1.00–1.01)	6.21 (5.37–7.18)	0.6
Suicide				
Swedish-born individuals	7.99 (7.38–8.65)	1.05 (1.05–1.06)	8.42 (7.78–9.11)	5.8
Refugees	7.40 (5.07–10.79)	1.01 (1.00–1.01)	7.45 (5.11–10.87)	0.8
DP				
Suicide attempt				
Swedish-born individuals	10.01 (9.68–10.36)	1.02 (1.02–1.03)	10.26 (9.92–10.61)	2.7
Refugees	6.06 (5.23–7.02)	1.03 (1.00–1.05)	6.22 (5.38–7.19)	3.0
Suicide				
Swedish-born individuals	8.37 (7.72–9.06)	1.02 (1.01–1.03)	8.56 (7.91–9.27)	2.6
Refugees	7.50 (5.13–10.97)	1.00 (0.97–1.04)	7.51 (5.15–10.95)	0.1

Direct effect: mental disorder → outcome. Indirect effect: mental disorder → mediator → outcome. Total effect: combined direct and indirect effect

^a^Adjusted for age, sex, educational level, family situation and place of residence

A small mediating effect of DP was observed (Table 4, lower part). When analyzing suicide attempt as outcome, in Swedish-born, the proportion of the total effect mediated by DP was 2.7% and in refugees, 3.0%. For refugees, the indirect effect was not statistically significant, though.

### Sensitivity analyses

In the sensitivity analysis where we excluded individuals who were granted residence permit on ‘humanitarian grounds’ or ‘in need of protection’, results were similar to the main analyses. Finally, separate analyses for both suicide attempt and suicide, and events coded as undetermined intent, generated similar estimates (data not shown).

## Discussion

### Key results

The present study of 163,160 refugees and 4,031,898 Swedish-born individuals examined the associations between mental disorders, work disability and suicide attempt and suicide. Our findings indicate that SA and DP were associated with an elevated risk of suicide attempt in individuals with and without mental disorders. In both populations, the highest risks of suicide attempt and suicide were observed in those treated for mental disorders, who were on SA or DP. In Swedish-born individuals, there was a synergy effect between mental disorders and SA and DP, respectively, with respect to suicidal behavior. Both SA and DP were found to mediate the studied associations in Swedish-born individuals, but not in refugees.

In line with earlier research [3–6], our results revealed slightly higher levels of mental disorders in refugees than in Swedish-born individuals. Exposure to traumatic life events in refugees together with post-migration stress has been suggested to explain the elevated psychiatric morbidity in this group [3, 42].

In general, refugees had a lower risk of both suicide attempt and suicide when compared to the Swedish-born population. Associations between refugee status and suicide attempt and suicide have rarely been investigated [8, 9]. Hence, our study is one of the first to examine associations between mental disorders and suicide attempt and suicide in refugees. There have been studies on immigrants (not discriminating between refugees and non-refugee migrants) and asylum seekers, where both higher and lower rates of suicide and suicide attempt have been reported [10, 11].

In a recent study, we investigated these associations in a similar cohort, but with focus on diagnosis-specific mental disorders [9]. This study showed that, for most specific disorders, the rates and risk for both suicide attempt and suicide in individuals with a mental disorder were lower in refugees compared to the Swedish-born population. Studies investigating these associations in general populations regardless of migration status have demonstrated that individuals suffering from mental disorders have a considerable higher risk for both suicide attempt and suicide compared to individuals without mental disorders [13, 14, 43–45].

The current study extends existing research by taking into account the role of WD in the relationship between mental disorders and suicidal behavior. To the best of our knowledge, this is the first study to examine these associations in a nationwide setting.

In both refugees and the Swedish-born population, we found a positive association between mental disorders and long-term SA and DP. These findings have been reported earlier not only for the general population [20–22], but also for immigrants (refugees and non-refugee migrants combined) [20].

Regardless of presence of mental disorder, we found elevated risk estimates of both suicide attempt and suicide in refugees and Swedish-born individuals, who were either on long-term SA or DP in 2005. Both long-term SA and DP have been identified as risk factors for suicidal behavior in the general population [25–27], although the mechanisms underlying these associations are not fully clear. Our findings show that, in Swedish-born individuals, the effect of mental disorder on suicidal behavior was partly modified by WD, such that those exposed to both had a multifold increased risk as compared to unexposed. In Swedish-born people, WD also appeared to mediate the association between mental disorders and suicidal behavior. These findings emphasize the need to take WD factors into account in a suicide risk assessment.

One possible mechanism is that mental disorders lead to WD [20–22], and WD in turn may lead to social isolation, which is one of the main risk factors associated with suicidal outcomes [32]. Being excluded from the labor market might also result in loss of meaning, change of life style and loneliness that can affect the risk of suicidal behavior [30]. WD may also be a marker of deteriorated health and functioning among those treated for a mental disorder that in turn may play an important role in explaining the association between mental disorders and suicidal behavior.

For refugees, the modifying and mediating role of WD was less clear. We found no statistically significant effect, which may among others be due to lack of power. Among refugees treated for mental disorder who were either long-term sickness absent or on DP, less than 15 were treated for suicide attempt, and less than 5 died by suicide. Another potential explanation to the non-significant effect of WD in refugees is that refugees might have lower eligibility to social security benefits because of less access to the social insurance system, e.g. through lack of information. Refugees might also be less eligible for SA, as those benefits require income from earlier work, and unemployment is more common among refugees [23]. Finally, it may very well be so that the effect of WD on the association between mental disorder and suicidal behavior, in fact, differs for refugees and Swedish-born individuals. It has, for instance, been demonstrated that there are differences in access to and acceptance of specialized health care related to mental disorders in refugees compared to the population in the host country in OECD countries [46, 47]. Also the mechanisms linking mental disorders and suicidal behavior might look different in refugees than in Swedish-born. Therefore, more research examining these pathways is needed to plan culturally sensitive and person-based suicide prevention strategies.

### Strengths and limitations

The study used large population-based registers with high completeness and validity, and basically no loss to follow-up. Furthermore, the large population size allowed for detailed analyses of SA and DP, and ability to adjust for a range of important confounders. Despite these strengths, our findings should be interpreted in the context of the following limitations: Relying solely on register data, we have likely captured the most severe cases of mental disorder, which in turn may lead to underreporting of less severe types of disorders and psychological distress, especially in refugees where the prevalence of psychological distress often is high, but at the same time, utilization of specialized health care tends to be lower [48]. This was also the case for suicide attempt, which only included cases registered in inpatient care, i.e. we are likely to have an underreporting of the less severe cases. The lack of data on mental disorders and work disability throughout the entire follow-up period might have led to over- or underestimation of the reported estimates. Furthermore, due to cultural and religious stigma, underreporting of suicide attempt and suicide may be especially prevalent in refugees from Islamic or Muslim majority countries. Another limitation is that the refugee population also included those granted residence permit ‘in need of protection’ and ‘humanitarian grounds’. It has been shown that the comorbidity in the latter group is considered to be much higher than in other groups who get residence permit in Sweden. This might have introduced a potential negative health selection in the refugee group in our study. However, in sensitivity analyses, where we included and excluded these individuals, there were no significant differences in the results (data not shown). Finally, the registers used do not hold information on the first 14 days of SA spells among employees.

## Conclusion

The results of this study showed that, compared to those with no mental disorder, both refugees and Swedish-born persons had an elevated risk for both suicide attempt and suicide. There was a strong interaction between mental disorders and WD for subsequent suicidal behavior in Swedish-born individuals. Moreover, the association was also mediated by WD in Swedish-born. Also for refugees without mental disorder, WD was a risk factor for subsequent suicide attempt and suicide. Particularly, for Swedish-born individuals with mental disorders, information on work disability is vital in a clinical suicide risk assessment.

## Electronic supplementary material

Below is the link to the electronic supplementary material.

Supplementary material 1 (DOCX 19 kb)
