# Supplementary material for: Mental disorders and suicidal behavior in refugees and Swedish-born individuals: is the association affected by work disability?
Source: Soc Psychiatry Psychiatr Epidemiol. 2020 Jan 2;55(8):1061–71. doi: 10.1007/s00127-019-01824-5 (PMC7395008; doi:10.1007/s00127-019-01824-5)
Supplement: Supplementary file 1 — Supplementary material 1 (DOCX 19 kb) [file 127_2019_1824_MOESM1_ESM.docx]

***Supplementary table 1.*** *Evaluation of synergy effects between mental disorders and sickness absence (SA), and disability pension (DP) respectively, and suicide attempt. Crude and multi-adjusted hazard ratios (HR) with 95% confidence intervals (CI).*

|  |  | **Swedish-born individuals** | | | | **Refugees** | | | | |
| --- | --- | --- | --- | --- | --- | --- | --- | --- | --- | --- |
| **Mental disorder** | **SA** | **Suicide attempt, N (column %)** | **Model 1^a^** | **Model 2^b^** | **Model 3^c^** | | **Suicide attempt, N (column %)** | **Model 1^a^** | **Model 2^b^** | **Model 3^c^** |
| No | No SA | 12,804 (0.4) | 1 (REF) | 1 (REF) | 1 (REF) | | 723 (0.5) | 1 (REF) | 1 (REF) | 1 (REF) |
|  | SA | 2,403 (0.7) | 2.04 (1.95-2.13) | 2.48 (2.37-2.59) | 2.29 (2.19-2.39) | | 97 (0.7) | 1.36 (1.10-1.68) | 1.73 (1.39-2.14) | 1.61 (1.29-1.99) |
| Yes | No SA | 3,978 (5.2) | 15.25 (14.72-15.81) | 8.93 (8.60-9.27) | 6.66 (6.40-6.94) | | 219 (3.4) | 6.82 (5.87-7.94) | 5.86 (5.03-6.83) | 4.20 (3.56-4.96) |
|  | SA | 755 (6.0) | 17.72 (16.46-19.07) | 15.56 (14.44-16.76) | 11.30 (10.48-12.19) | | 17 (2.7) | 5.26 (3.25-8.51) | 5.71 (3.52-9.25) | 3.93 (2.41-6.39) |
| *Synergy Index* |  |  | *1.09 (1.01-1.19)* | *1.55 (1.42-1.68)* | *1.48 (1.36-1.61)* | |  | *0.69 (0.38-1.26)* | *0.84 (0.46-1.53)* | *0.77 (0.40-1.49)* |
|  |  |  |  |  |  | |  |  |  |  |
| **Mental disorder** | **DP** |  |  |  |  | |  |  |  |  |
| No | No DP | 15,112 (0.4) | 1 (REF) | 1 (REF) | 1 (REF) | | 811 (0.5) | 1 (REF) | 1 (REF) | 1 (REF) |
|  | DP | 95 (1.8) | 4.95 (4.05-6.06) | 3.45 (2.82-4.23) | 3.15 (2.57-3.86) | | <10^d^ | 2.26 (1.17-4.35) | 2.67 (1.38-5.18) | 2.32 (1.20-4.51) |
| Yes | No DP | 4,533 (5.2) | 14.08 (13.62-14.55) | 8.73 (8.43-9.03) | 6.50 (6.25-6.75) | | 225 (3.3) | 6.45 (5.56-7.47) | 5.52 (4.75-6.41) | 3.98 (3.38-4.68) |
|  | DP | 200 (10.9) | 31.35 (27.27-36.04) | 14.74 (12.81-16.96) | 10.27 (8.91-11.83) | | 11 (4.2) | 8.23 (4.54-14.92) | 8.33 (4.58-15.13) | 5.46 (2.99-9.99) |
| *Synergy Index* |  |  | *1.78 (1.52-2.08)* | *1.35 (1.14-1.59)* | *1.21 (1.02-1.45)* | |  | *1.08 (0.53-2.21)* | *1.18 (0.57-2.48)* | *1.04 (0.46-2.35)* |

^a^ Model 1: Crude

^b^ Model 2: Adjusted for age, sex, educational level, family situation and place of residence

^c^ Model 3: Model 2 with additional adjustments for history of suicide attempt and somatic comorbidity

^d^ For ethical reasons i.e. the risk of identification of individuals, if the number of events is <10, it is not reported

***Supplementary table 2.*** *Evaluation of synergy effects between mental disorders and sickness absence (SA), and disability pension (DP) respectively, and suicide. Crude and multi-adjusted hazard ratios (HR) with 95% confidence intervals (CI).*

|  |  | **Swedish-born individuals** | | | | **Refugees** | | | |
| --- | --- | --- | --- | --- | --- | --- | --- | --- | --- |
| **Mental disorder** | **SA** | **Suicide, N (column %)** | **Model 1^a^** | **Model 2^b^** | **Model 3^c^** | **Suicide, N (column %)** | **Model 1^a^** | **Model 2^b^** | **Model 3^c^** |
| No | No SA | 3,259 (0.1) | 1 (REF) | 1 (REF) | 1 (REF) | 90 (0.1) | 1 (REF) | 1 (REF) | 1 (REF) |
|  | SA | 552 (0.2) | 1.83 (1.67-2.01) | 2.09 (1.91-2.29) | 2.04 (1.86-2.24) | 20 (0.1) | 2.24 (1.38-3.64) | 2.50 (1.53-4.10) | 2.33 (1.42-3.82) |
| Yes | No SA | 626 (0.8) | 9.21 (8.45-10.03) | 7.85 (7.19-8.57) | 6.72 (6.12-7.38) | 32 (0.5) | 7.90 (5.28-11.83) | 8.02 (5.33-12.08) | 6.02 (3.85-9.43) |
|  | SA | 139 (1.1) | 12.35 (10.42-14.64) | 11.60 (9.78-13.77) | 9.78 (8.22-11.65) | <10^d^ | 9.83 (3.61-26.76) | 10.63 (3.89-29.07) | 8.56 (3.11-23.60) |
| *Synergy Index* |  |  | *1.26 (1.03-1.53)* | *1.33 (1.09-1.63)* | *1.30 (1.06-1.59)* |  | *1.08 (0.34-3.42)* | *1.13 (0.36-3.53)* | *1.19 (0.37-3.85)* |
|  |  |  |  |  |  |  |  |  |  |
| **Mental disorder** | **DP** |  |  |  |  |  |  |  |  |
| No | No DP | 3,790 (0.1) | 1 (REF) | 1 (REF) | 1 (REF) | 110 (0.1) | 1 (REF) | 1 (REF) | 1 (REF) |
|  | DP | 21 (0.4) | 4.35 (2.84-6.69) | 3.48 (2.27-5.36) | 3.32 (2.16-5.11) | 0 (0.0) | N/A | N/A | N/A |
| Yes | No DP | 734 (0.8) | 8.87 (8.19-9.60) | 7.68 (7.08-8.33) | 6.54 (6.00-7.13) | 34 (0.5) | 7.09 (4.83-10.41) | 7.20 (4.87-10.63) | 5.49 (3.59-8.39) |
|  | DP | 31 (1.7) | 18.21 (12.79-25.92) | 12.96 (9.09-18.49) | 10.29 (7.19-14.72) | <10^d^ | 10.87 (2.68-44.0) | 10.72 (2.63-43.65) | 6.78 (1.62-28.42) |
| *Synergy Index* |  |  | *1.53 (1.02-2.31)* | *1.31 (0.86-1.99)* | *1.18 (0.77-1.83)* |  | *1.94 (0.39-9.58)* | *1.87 (0.38-9.28)* | *1.66 (0.30-9.24)* |

^a^ Model 1: Crude

^b^ Model 2: Adjusted for age, sex, educational level, family situation and place of residence

^c^ Model 3: Model 2 with additional adjustments for history of suicide attempt and somatic comorbidity

^d^ For ethical reasons i.e. the risk of identification of individuals, if the number of events is <10, it is not reported
